# Asiatic Acid Inhibits Nasopharyngeal Carcinoma Cell Viability and Migration via Suppressing STAT3 and Claudin-1

**DOI:** 10.3390/ph16060902

**Published:** 2023-06-19

**Authors:** Supitchaya Pantia, Thaned Kangsamaksin, Tavan Janvilisri, Waraporn Komyod

**Affiliations:** 1 Graduate Program in Molecular Medicine, Faculty of Science, Mahidol University, Bangkok 10400, Thailand; supitchaya.pantia@gmail.com; 2 Department of Biochemistry, Faculty of Science, Mahidol University, Bangkok 10400, Thailand; thaned.kan@mahidol.edu (T.K.); tavan.jan@mahidol.ac.th (T.J.)

**Keywords:** nasopharyngeal carcinoma, asiatic acid, STAT3, claudin-1, EMT, apoptosis

## Abstract

Nasopharyngeal carcinoma (NPC) is a prevalent cancer in Southeast Asia, but effective treatment options remain limited, and chemotherapy has a high resistance rate. Asiatic acid (AA), a triterpenoid found in *Centella asiatica*, has shown anticancer activity in various cancers. Therefore, this study aims to investigate the anticancer effects and mechanisms of AA in NPC cell lines. The effects of AA on NPC cytotoxicity, apoptosis, and migration were determined in TW-01 and SUNE5-8F NPC cell lines. Western blot analysis was performed to evaluate the protein expression levels affected by AA. The role of AA in proliferation and migration was investigated in STAT3 and claudin-1 knockdown cells. AA inhibited NPC cell viability and migration and induced cell death by increasing cleaved caspase-3 expression. Moreover, AA inhibited STAT3 phosphorylation and reduced claudin-1 expression in NPC cells. Although knockdown of STAT3 or claudin-1 slightly reduced cell viability, it did not enhance the anti-proliferative effect of AA. However, knockdown of STAT3 or claudin-1 increased the anti-migratory effect of AA in NPC cells. These results suggest that AA can be a promising candidate for drug development against NPC.

## 1. Introduction

Nasopharyngeal carcinoma (NPC) is a significant public health concern in Asia, particularly in Southeast Asia, with high incidence and mortality rates [1]. The pathogenesis of NPC is multifactorial, involving genetic, environmental, and viral factors such as Epstein–Barr virus (EBV) infection, dietary habits, and exposure to chemical agents [2]. Current treatment options for NPC are limited, with surgery being difficult due to the location of the tumor [3]. Radiotherapy is the primary treatment for NPC, with chemotherapy as a complementary option for advanced-stage cases [4]. Cisplatin-based drugs are the most effective chemotherapy for metastatic NPC; however, their use is limited due to their significant side effects, including renal, hepatic, and cardiovascular toxicity [5,6]. Therefore, the identification and development of alternative therapies with improved safety profiles are necessary to improve the management of NPC.

Plant-derived compounds are currently being studied for their potential use in cancer treatment. Among these natural compounds, triterpenoids have shown promising anticancer activities. Asiatic acid (AA), a pentacyclic triterpenoid found in *Centella asiatica*, a tropical plant used in traditional medicine and as a dietary supplement, has been extensively studied in recent years [7]. AA exhibits anti-inflammatory [8] and neuroprotective functions [9] and is involved in various molecular pathways. In cancer research, AA has been shown to inhibit proliferation and induce apoptosis in HepG2 cells by enhancing p53 expression [10]. In breast cancer cells, AA inhibits cell proliferation by inducing cell cycle arrest at the S-G2/M phase [11]. Additionally, AA has demonstrated anticancer activity against ovarian cancer by inhibiting the PI3K/Akt/mTOR pathway [12] and promoting AA-induced apoptosis in NSCLC cells by upregulating miR-1290 [13]. In skin cancer cells, AA increases the ROS level, leading to cancer cell death [14]. AA has also been reported to block angiogenesis in glioblastomas [15] and activate the mitochondrial death cascade in colon cancer [16]. Recent studies have shown that AA can downregulate anti-apoptotic genes BCL-2 and survivin/BIRC5 [17], demonstrating its anticancer activity against human cholangiocarcinoma cells. Despite these findings, the effect of AA on human NPC remains unclear and requires further investigation.

The JAK/STAT pathway has been associated with many types of cancer, making it a potential target for anticancer therapy [18]. STAT3 activation has been shown to play a role in cancer cell proliferation, immune invasion [18], apoptosis [19], angiogenesis [20], and metastasis [21]. In gastrointestinal cancer, STAT3 has been found to promote epithelial–mesenchymal transition (EMT), the initial step of cancer metastasis [22]. Constitutive activation of STAT3 has also been found in over 75% of NPC tumors [23]. The overexpression of activated STAT3 is associated with the advanced stage of NPC [24], and the activation of STAT3 directly contributes to the cellular invasiveness of NPC [25]. In light of these findings, targeting STAT3 has emerged as a promising strategy for NPC therapy. Additionally, evidence suggests that asiatic acid may have an effect on the STAT3 pathway. Studies have shown that asiatic acid derivatives can inhibit gastric cancer cell proliferation and invasion by suppressing JAK2 and STAT3 activation [26]. Furthermore, AA has been found to inhibit pro-inflammatory cytokine secretion (TNF-α and IL-6) by suppressing NF-kB, STAT3, and ERK pathways [27].

EMT is a process by which epithelial cells lose their localized functions and acquire migratory mesenchymal characteristics. This process is characterized by the breakdown of cell–cell junctions, disruption of the basement membrane, and changes in the expression of EMT markers, for example, the downregulation of epithelial markers such as E-cadherin and the upregulation of mesenchymal markers such as N-cadherin [28]. The regulation of EMT involves multiple signaling pathways, including the IL-6/STAT3 pathway, which controls the expression of key transcription factors TWIST1 and SNAIL1 [29]. Asiatic acid has been reported to have anticancer effects in many types of cancer, but its effects on NPC remain unclear. Therefore, in this study, we aimed to investigate the impact of AA on cytotoxicity, apoptosis, migration, and the underlying molecular mechanisms in NPC cell lines. Our research postulates that AA exhibits cytotoxicity against nasopharyngeal carcinoma (NPC) cell lines, and its impact is discernibly more selective toward cancer cells rather than normal cells. Furthermore, we hypothesize that AA potentially induces cellular apoptosis while concurrently impeding cell migration, a pivotal mechanism associated with the progression of cancer. Additionally, our investigation delves into the effects of AA on STAT3 and epithelial–mesenchymal transition (EMT) pathways, with the aim of proposing potential molecular targets that may be modulated by this compound.

## 2. Results

### 2.1. Cytotoxicity of Asiatic Acid to NPC and Normal Cells

**Figure 1:**
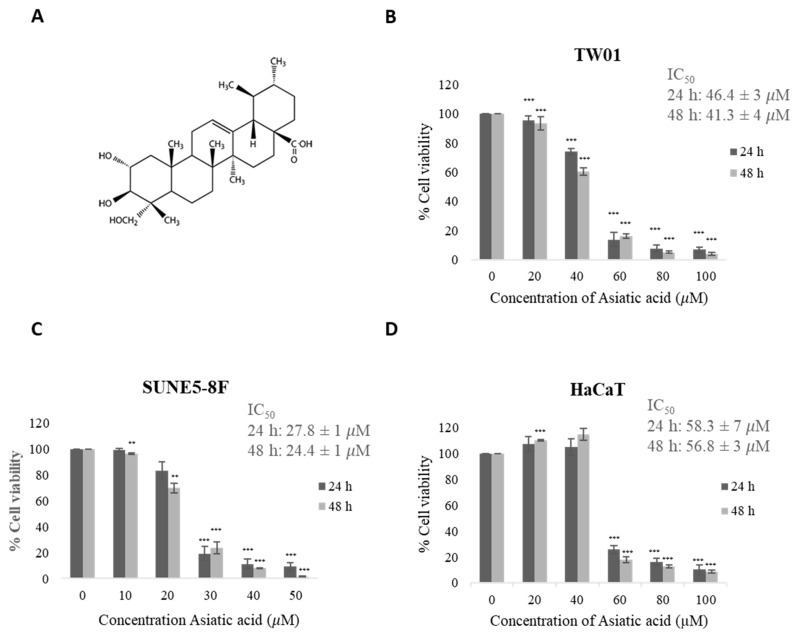
Effect of asiatic acid on cell viability. (**A**) Structure of asiatic acid. Cytotoxic effect of asiatic acid on two nasopharyngeal carcinoma cell lines [30], (**B**) TW-01, (**C**) SUNE5-8F, and (**D**) normal keratinocyte cell line, HaCaT cells, were determined by MTT assay. Cells were treated with various concentrations of asiatic acid (0, 10, 20, 30, 40, and 50 μM for SUNE5-8F and 0, 20, 40, 60, 80, and 100 μM for TW01 and HaCaT) for 24 h and 48 h. Final concentration of DMSO was 0.1% in all concentrations of asiatic acid. Data represent the mean ± SE of three independent experiments, ** *p* ≤ 0.01, *** *p* ≤ 0.001 compared with the untreated control.

Figure 1A depicts the structure of asiatic acid (AA) [30]. The MTT assay was conducted to measure the cell viability of two NPC cell lines (TW01 and SUNE5-8F) and a keratinocyte cell line (HaCaT) treated with various concentrations of AA for 24 and 48 h. The results showed that AA significantly reduced cell viability in a dose-dependent manner compared with DMSO-treated cells as a control (Figure 1B,C). However, HaCaT cells exhibited less cytotoxicity compared with NPC cells (Figure 1D). The IC_50_ values of AA at 24 h were 46.4 ± 3, 27.8 ± 1, and 58.3 ± 7 μM for TW01, SUNE5-8F, and HaCaT cells, respectively. At 48 h, the IC50 values were 41.3 ± 4, 24.4 ± 1, and 56.8 ± 3 μM for TW01, SUNE5-8F, and HaCaT cells, respectively. The selectivity index values for TW01 and SUNE5-8F at 24 h were determined to be 1.256 and 2.097, respectively. The selectivity index of >1 indicated that AA is more effective against cancer cells compared with its toxicity against normal cells. These findings suggest the potential of AA as a cytotoxic agent against NPC cells.

### 2.2. Asiatic Acid Increased Cleaved Caspase-3 Expression

**Figure 2:**
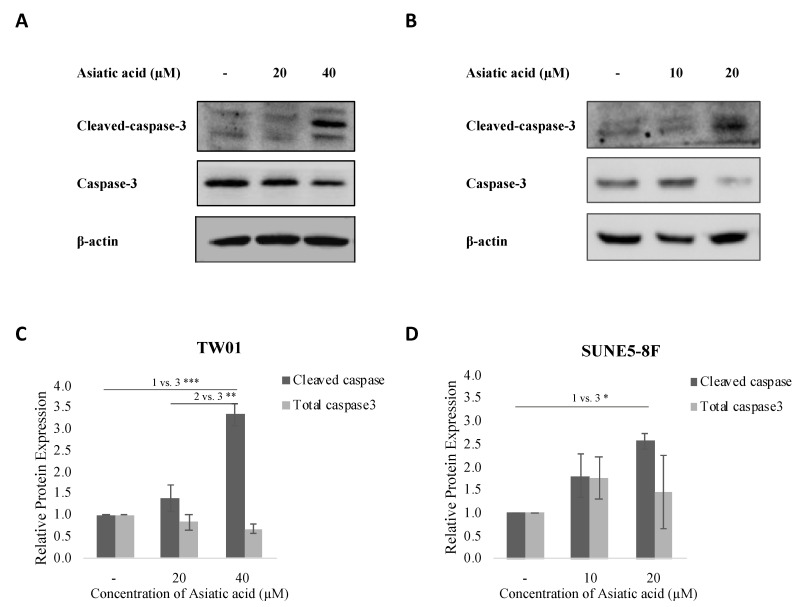
Effect of asiatic acid on cell apoptosis of nasopharyngeal carcinoma cells. (**A**) TW01 and (**B**) SUNE5-8F cells were treated with asiatic acid at concentrations below the IC50 of each cell line as indicated for 24 h. Western blot analysis was performed to examine the levels of cleaved caspase-3 protein expression in TW01 and SUNE5-8F cells. β-actin was used as a loading control. (**C**,**D**) Densitometry analysis of protein expression was quantified using ImageJ software. Data represented as mean ± SE from three independent experiments. * *p* ≤ 0.05, ** *p* ≤ 0.01, *** *p* ≤ 0.001 using one-way ANOVA followed by Tukey’s honestly significant difference test.

In this study, we investigated the effect of asiatic acid on cell death in NPC cells. TW01 and SUNE5-8F cells were treated with asiatic acid at concentrations below the IC50 value for each cell line (0, 20, and 40 μM in TW01 and 0, 10, and 20 μM in SUNE5-8F) for 24 h. The expression of cleaved caspase-3 was analyzed by Western blotting. Our results showed that asiatic acid significantly induced the expression of cleaved caspase-3 at 40 and 20 μM in TW01 and SUNE5-8F cells, respectively (Figure 2). These findings suggest that asiatic acid might induce cell death by activating caspase-3 in NPC cell lines.

### 2.3. Asiatic Acid Inhibited NPC Cell Migration

**Figure 3:**
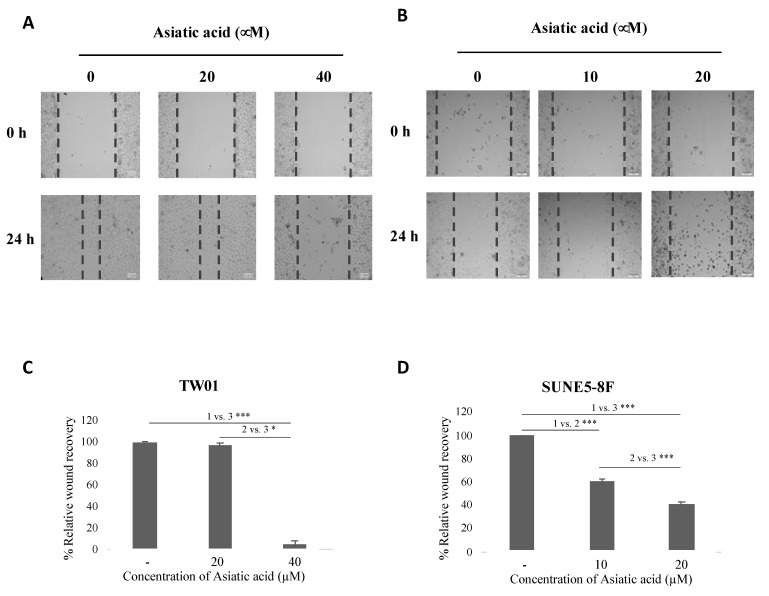
Effect of asiatic acid on cell migration of nasopharyngeal carcinoma cells. (**A**) TW01 and (**B**) SUNE5-8F cells were seeded in a 24-well plate for 24 h. Wound was created by a 200 μL pipette tip and then treated with AA at indicated concentrations for 24 h. Images of migration were observed under a light microscope (10× magnification) at 0 and 24 h. (**C**,**D**) Wound areas were analyzed by ImageJ software and reported as relative wound recovery. Data represented as mean ± SE from three independent experiments. * *p* ≤ 0.05 and *** *p* ≤ 0.001 using one-way ANOVA followed by Tukey’s honestly significant difference test.

To evaluate the effect of asiatic acid on NPC cell migration, we performed a wound healing assay. TW01 and SUNE5-8F cells were treated with different concentrations of asiatic acid (0, 20, and 40 μM in TW01 and 0, 10, and 20 μM in SUNE5-8F). Our results showed that asiatic acid inhibited cell migration in both NPC cell lines. In TW01 cells, cell migration was significantly inhibited at 40 μM of asiatic acid (Figure 3A,C). Similarly, treatment with 20 μM of asiatic acid inhibited cell migration by around 50% in SUNE5-8F cells (Figure 3B,D). These results suggest that asiatic acid exerts anti-migratory activity in NPC cells.

### 2.4. Asiatic Acid Inhibited STAT3 Pathway in NPC Cell Lines

**Figure 4:**
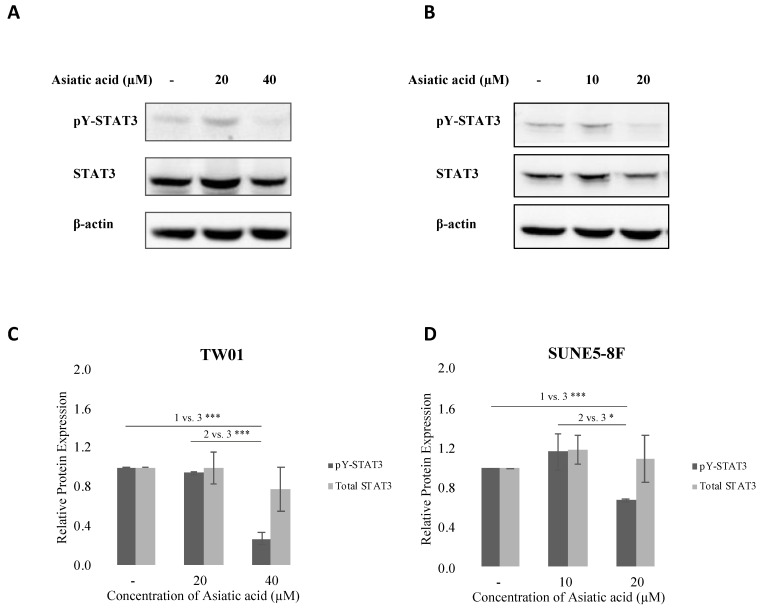
Effect of asiatic acid on STAT3 phosphorylation in nasopharyngeal carcinoma cells. (**A**) TW01 and (**B**) SUNE5-8F cells were treated with asiatic acid at the indicated concentrations for 24 h. Western blot analysis was performed to examine the levels of phospho-STAT3 protein expression in TW01 and SUNE5-8F cells. β-actin was used as a loading control. (**C**,**D**) Densitometry analysis of protein expression was quantified using ImageJ software. Data represented as mean ± SE from three independent experiments. * *p* ≤ 0.05 and *** *p* ≤ 0.001 using one-way ANOVA followed by Tukey’s honestly significant difference test.

The STAT3 pathway plays a critical role in NPC cell proliferation, migration, and survival. Therefore, we examined the effect of asiatic acid on STAT3 phosphorylation in NPC cells. TW01 and SUNE5-8F cells were treated with various concentrations of asiatic acid for 24 h. Our results showed that asiatic acid inhibited STAT3 phosphorylation, with the inhibition observed at 40 μM and 20 μM concentrations in TW01 and SUNE5-8F cells, respectively (Figure 4A,B). These findings suggest that asiatic acid exerts anticancer activities by suppressing the STAT3 pathway in NPC cell lines.

### 2.5. Asiatic Acid Modulated EMT Markers Expression in NPC Cell Lines

**Figure 5:**
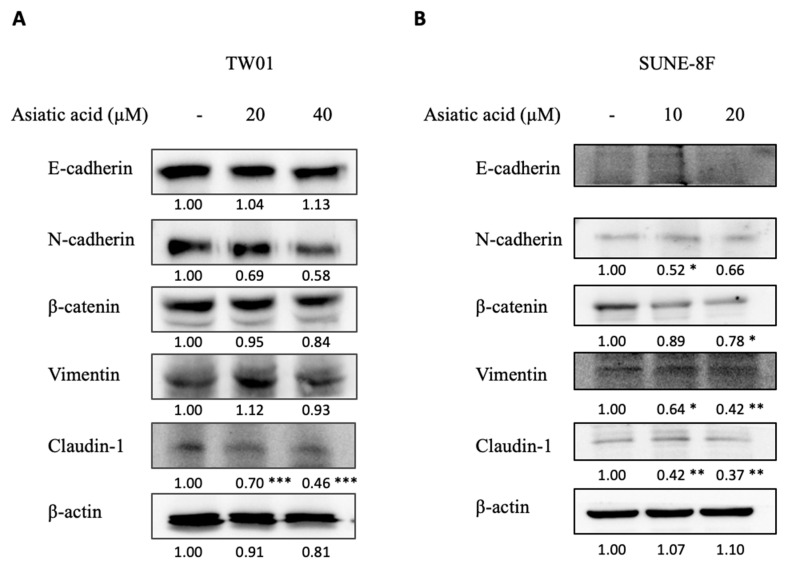
Effect of asiatic acid on EMT protein expression in nasopharyngeal carcinoma cells. (**A**) TW01 and (**B**) SUNE5-8F cells were treated with asiatic acid at the indicated concentrations for 24 h. Western blot analysis was performed to examine the levels of EMT protein expression in TW01 and SUNE5-8F cells. β-actin was used as a loading control. Data represented as mean ± SE from three independent experiments. * *p* ≤ 0.05, ** *p* ≤ 0.01, *** *p* ≤ 0.001 compared with control using one-way ANOVA followed by Tukey’s honestly significant difference test.

Epithelial–mesenchymal transition (EMT) is a crucial process involved in cancer cell migration and metastasis. Previous findings demonstrated that AA inhibited NPC cell migration. In this study, we aimed to investigate whether AA alters the expression of EMT markers in NPC cells. The results of the Western blot analysis revealed that AA exerted different effects on the EMT markers of each cell line (Figure 5A,B). AA slightly decreased mesenchymal markers N-cadherin and β-catenin in both NPC cell lines and vimentin in SUNE5-8F. Interestingly, AA significantly reduced claudin-1 expression in both cell lines. These findings suggest that AA might exert its function via downregulating EMT markers.

### 2.6. Knockdown of STAT3 or Claudin-1 Reduced NPC Cell Viability

**Figure 6:**
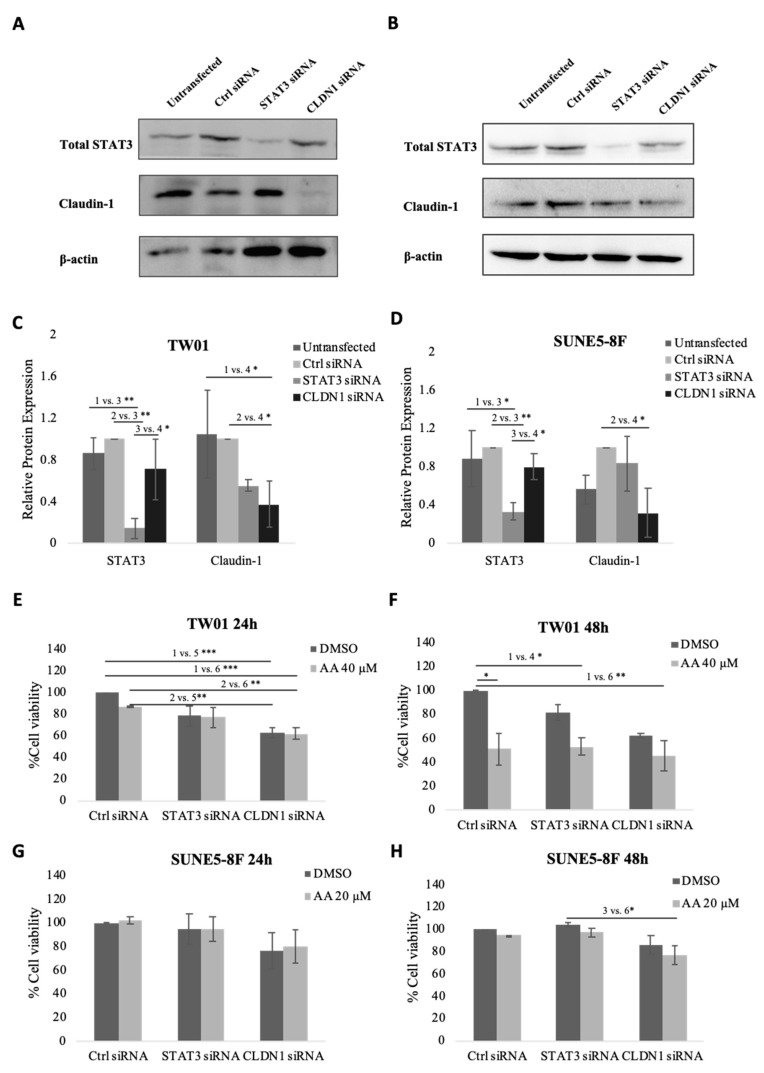
Effect of knockdown STAT3 or claudin-1 on cell viability of nasopharyngeal carcinoma cells. (**A**) TW01 and (**B**) SUNE5-8F cells were transfected with 10 nM of control, STAT3, or claudin-1 siRNA for 48 h. Knockdown efficiency was analyzed by Western blot and quantified by ImageJ (**C**,**D**). Cytotoxic effect of AA in TW01 (**E**,**F**) and SUNE5-8F (**G**,**H**) knockdown cells was determined by MTT assay. NPC cells were transfected with control, STAT3, or claudin-1 siRNA for 24 h. Then cells were seeded to a 96-well plate and allowed to attach for 24 h. After that, cells were treated with AA at a concentration below IC50 of each cell line for 24 h and 48 h. Data represented as mean ± SE from three independent experiments. * *p* ≤ 0.05, ** *p* ≤ 0.01, *** *p* ≤ 0.001 using one-way ANOVA followed by Tukey’s honestly significant difference test.

In previous experiments, we observed that AA suppressed STAT3 phosphorylation and claudin-1 expression. To assess whether these genes are necessary for the anticancer effect of AA, we knocked down STAT3 and claudin-1 in TW01 and SUNE5-8F cells using siRNA. Figure 6A–D demonstrate the knockdown efficiency of STAT3 and CLDN1 in both cell lines. Suppression of STAT3 slightly reduced the cell viability of TW01 cells but was not significant in SUNE5-8F. On the other hand, suppression of CLDN1 significantly reduced cell viability in TW01 (Figure 6E,F) and slightly reduced cell viability in SUNE5-8F (Figure 6G,H) compared with control siRNA. However, the anti-proliferative effect of asiatic acid was not enhanced by silencing CLDN1. These results indicate that CLDN1 plays a crucial role in NPC cell viability but not in the anti-proliferative activity of asiatic acid.

### 2.7. Knockdown of STAT3 or Claudin-1 Inhibited Migration in TW01

**Figure 7:**
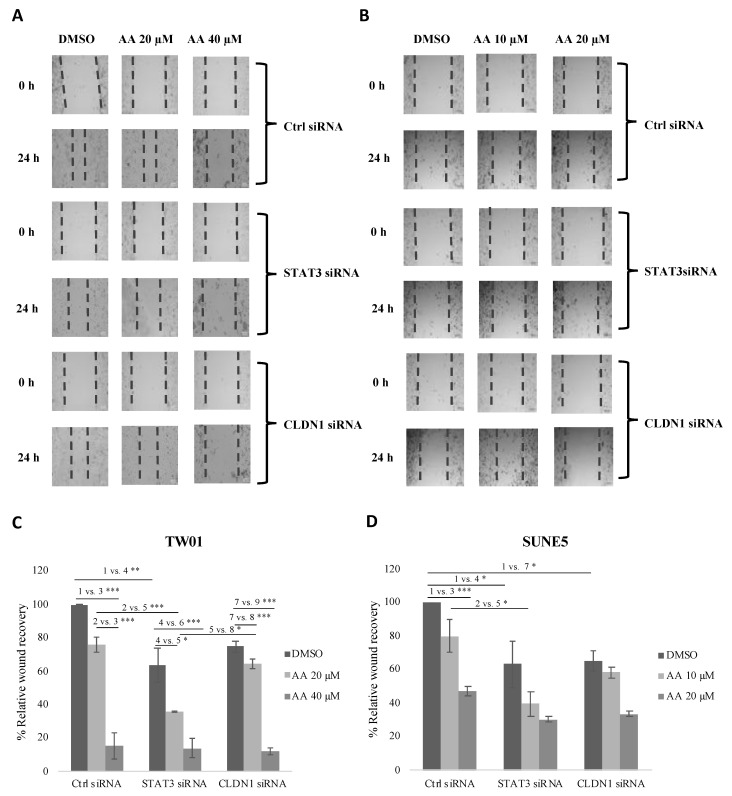
Effect of asiatic acid on cell migration in STAT3 or claudin-1 knockdown cells. (**A**) TW01 and (**B**) SUNE5-8F cells were knocked down with 10 nM of control, STAT3, or claudin-1 siRNA for 24 h, then cells were seeded in a 24-well plate for 24 h, and the wound was created by a 200 μL pipette tip and then treated with AA for 24 h. Images of migration were observed under a light microscope (10× magnification) at 0 and 24 h. Wound areas were analyzed by ImageJ software and reported as relative wound recovery (%) for TW01 (**C**) and SUNE5-8F (**D**). Data represented as mean ± SE from three independent experiments. * *p* ≤ 0.05, ** *p* ≤ 0.01, *** *p* ≤ 0.001 using one-way ANOVA followed by Tukey’s honestly significant difference test.

We also examined the effect of STAT3 and claudin-1 knockdown on the anti-migratory effect of asiatic acid. Knockdown of STAT3 and claudin-1 inhibited the migration of TW01 (Figure 7A) and SUNE5-8F (Figure 7B). Furthermore, the anti-migratory effect of AA was increased in STAT3 and claudin-1 knockdown cells. Our results suggest that STAT3 and claudin-1 mediate the anti-migratory effect of asiatic acid.

## 3. Discussion

The anticancer properties of AA have been extensively demonstrated in a range of cancers including breast, ovarian, colon, hepatoma, glioblastoma, and cholangiocarcinoma (10–12, 15–17). Despite this, the effects of AA and its molecular mechanisms against NPC remain unknown. Thus, the purpose of this study is to examine the cytotoxicity, anti-migratory effects, and protein marker expression underlying the anticancer effect of AA in NPC cell lines.

The findings of this study indicate that AA exhibits anticancer activity against NPC. Our results reveal that AA induced cell cytotoxicity in a dose-dependent manner, with a higher IC_50_ in normal keratinocytes (HaCaT cell line) than in NPC cell lines (Figure 1). This suggests that NPC cells are more susceptible to AA than normal cells. The selectivity index values of TW01 and SUNE5-8F at 24 h were 1.256 and 2.097, respectively. The favorable SI values (>1.0) indicate that AA exhibits a higher efficacy against tumor cells compared with its toxicity against normal cells. However, further studies are required to establish the appropriate range of selectivity index for AA in different types of cancer, including NPC. Our results strongly support the anticancer activity of AA against NPC cell lines.

Furthermore, we observed an upregulation of cleaved caspase-3 protein expression in response to AA treatment (Figure 2), which is consistent with a previous study demonstrating apoptosis induction by AA in cisplatin-resistant NPC [31]. However, additional investigations, such as Annexin/PI flow cytometry or immunohistochemistry targeting other apoptosis markers, are necessary to confirm that AA induces NPC cell death through the apoptosis pathway in our specific model.

Additionally, AA has been reported to inhibit cell migration in colon cancer through the PI3K/Akt/mTOR/p70S6K and epithelial–mesenchymal transition (EMT) pathways [32]. In renal cancer, AA suppressed cell migration and invasion by downregulating the mRNA and protein expression of MMP-15 (matrix metallopeptidase-15) [33]. Our results demonstrate that AA also inhibits migration in TW01 and SUNE5-8F cell lines (Figure 3). Importantly, the anti-migratory effect of AA on both cell lines is independent of reduced cell viability at the concentrations tested.

The JAK/STAT pathway has been implicated in various types of cancers. Numerous studies have reported persistent STAT activation in human cancers, including blood tumors and solid tumors such as leukemia, myeloma, lymphoma, melanoma, lung cancer, and prostate cancer [18], highlighting the potential of targeting STATs for anticancer therapy. Elevated levels of JAK2 and STAT3 protein expression have been observed in NPC tissues [34]. In addition, in more than 75% of NPC tumors, STAT3 has been constitutively activated, directly contributing to the cancer’s invasiveness [23,25]. Therefore, inhibiting the JAK/STAT3 pathway can be an effective therapeutic approach for NPC. Evidence has shown that asiatic acid can affect the STAT3 pathway. For instance, asiatic acid derivatives have been found to suppress JAK2 and STAT3 activation, inhibiting gastric cancer cell proliferation and invasion [26]. Additionally, AA has been shown to inhibit pro-inflammatory cytokine secretion by inhibiting the NF-κB, STAT3, and ERK pathways [27]. Our study demonstrates that AA can inhibit STAT3 phosphorylation in NPC cell lines (Figure 4), indicating that AA may exert its anticancer effects by inhibiting the STAT3 pathway. Furthermore, our study demonstrates that AA effectively inhibits both constitutive STAT3 phosphorylation and IL-6-induced STAT3 phosphorylation in NPC cells (Figure S1).

In addition to exploring the influence of AA on the JAK/STAT3 pathway, we conducted investigations into its effects on epithelial–mesenchymal transition (EMT) markers. EMT refers to a cellular process wherein an epithelial cell undergoes a phenotypic transition, acquiring mesenchymal characteristics and the ability to migrate away from its original location [35]. Previous studies have examined the impact of AA on EMT in lung cancer cells. Notably, AA treatment resulted in the increased expression of E-cadherin and decreased expressions of snail, N-cadherin, vimentin, and β-catenin [36]. While EMT is a crucial process in cancer progression, our results show that AA has little effect on the majority of EMT marker proteins, including E-cadherin, N-cadherin, β-catenin, and vimentin. However, AA significantly reduced the expression of claudin-1, an epithelial marker, in TW01 and SUNE5-8F cell lines (Figure 5). Claudin is a tight junction protein that regulates the cell–cell interaction across cell membranes. Various types of claudin protein have been dysregulated in different cancers and are involved in cancer metastasis. In NPC, elevated levels of claudin-1 have been linked to lymph node metastasis and clinical staging. Claudin-1 promotes cell proliferation, migration, and invasion of NPC cells by activating the Wnt/β-catenin signaling pathway [37]. Therefore, the downregulation of claudin-1 may be one of the mechanisms by which AA exerts its anticancer effects in NPC.

In order to establish the molecular mechanism of AA, we utilized siRNA to transiently knock down the expression of STAT3 and claudin-1 in NPC cells. This allowed us to investigate whether the absence of these proteins enhances the effect of AA. Our findings indicate that the suppression of both STAT3 and claudin-1 significantly inhibited NPC cell viability (Figure 6). However, while the knockdown of STAT3 did not enhance the effect of AA, the silencing of claudin-1 markedly amplified the effect of AA in TW01 cells after 48 h (Figure 6F). These results confirm the important role of claudin-1 in NPC cell viability. Additionally, we observed that the absence of both STAT3 and claudin-1 increased the anti-migratory effect of AA in NPC cells (Figure 7). These findings suggest that both STAT3 and claudin-1 are involved in the cell migration of NPC. Nonetheless, further research is required to investigate the correlation between STAT3 and claudin-1 in NPC.

In conclusion, our study provides compelling evidence for the potential of AA as a promising therapeutic option for nasopharyngeal carcinoma (NPC). We have demonstrated its multifaceted anticancer effects, including the reduction of cell viability, inhibition of cell migration, and induction of cell death. Notably, AA exhibits a dose-dependent decrease in NPC cell viability while displaying a more selective cytotoxicity toward cancer cells compared with normal cells. Moreover, AA treatment leads to the upregulation of cleaved caspase-3 protein expression, a crucial marker of cell apoptosis. Additionally, our findings highlight the inhibitory effect of AA on NPC cell migration, a pivotal process in cancer metastasis. Furthermore, we have unveiled the suppressive impact of AA on STAT3 activation and claudin-1 expression, two key molecules implicated in the pathogenesis of NPC. These novel insights shed light on the potential therapeutic targets of AA in the context of NPC.

## 4. Materials and Methods

### 4.1. Materials

Asiatic acid (97%) (#546712, Sigma-Aldrich, St Louis, MO, USA) was prepared as a stock solution of 100 mM by dissolving 4.887 mg of asiatic acid in 100 μL of dimethyl sulfoxide (DMSO). The solution was stored in the dark at −20 °C until use. All experiments contained <0.1% (*v*/*v*) DMSO. Cell culture reagents including Roswell Park Memorial Institute medium (RPMI) 1640 (#31800022), Dulbecco’s modified Eagle medium (DMEM; #31600034), fetal bovine serum (FBS; #10270098), trypsin–EDTA (0.25%), phenol red (#25200072), and penicillin–streptomycin (10,000 U/mL) (#15140122) were from Thermo Fisher Scientific (Waltham, MA, USA).

### 4.2. Cell Culture

NPC cell lines in this study included SUNE 5-8F (kindly obtained from Prof. Qingling Zhang, Southern Medical University) and TW01 (kindly gifted by Prof. C-T Lin, National Taiwan University). An immortalized human keratinocyte cell line, HaCaT cells (from ATCC), was also included in the study. SUNE 5-8F and HaCaT cells were cultured in RPMI 1640 supplemented with 10% FBS and 100 U/mL penicillin and 100 µg/mL streptomycin, incubated at 37 °C with 5% CO_2_. TW01 cells were cultured in DMEM supplemented with 10% FBS and 100 U/mL of penicillin and 100 µg/mL of streptomycin at 37 °C with 5% CO_2_. All cell lines were sub-cultured at 80–90% confluence, and the media were replaced every 48 h.

### 4.3. Cell Viability Assay

Cell viability was assayed by the MTT (3-(4,5-dimethylthiazol-2-yl)-2,5-diphenyltetrazolium bromide) method. About 5 × 10^3^ cells/well were seeded in a 96-well plate and incubated for 24 h. The cells were treated with various concentrations of asiatic acid (TW01: 0, 20, 40, 60, 80, and 100 μM; SUNE5-8F: 0, 10, 20, 30, 40, and 50 μM) in 1% FBS-containing media for 24 and 48 h. After incubation, the medium was removed, and an MTT reagent (0.5 mg/mL final concentration) was added to each well for 3 h. Formazan crystals were dissolved by adding DMSO, and the absorbance of formazan dye was measured at 540 nm using a microplate reader. The percentage of cell viability was calculated as the ratio of the absorbance of the treated cells to the absorbance of the untreated cells. The experiments were performed in triplicate for each condition and repeated three times. The half-maximal inhibitory concentration (IC_50_) was calculated by the Chou–Talalay method [38]. The selectivity of AA against each NPC cell line relative to normal cells was employed by the following equation: SI (selectivity Index) = IC_50_ of non-cancerous cells/IC_50_ of cancer cells [39].

### 4.4. Western Blot Analysis

The cells were lysed with HEPES lysis buffer supplemented with a protease inhibitor cocktail (#HY-K0010, MedChemExpress, Monmouth Junction, NJ, USA) and Na_3_VO_4_, a phosphatase inhibitor. The protein concentration was determined using the Bradford protein assay (Bio-Rad, Hercules, CA, USA). The proteins were separated by 8–12% SDS-PAGE and transferred onto the PVDF membrane. The membranes were blocked with 5% BSA in TBS-N for 1 h with these following primary antibodies: pY-STAT3 (Y705) (#9131S), caspase-3 (#9662S), vimentin (D21H3) (#5741T), E-cadherin (24E10) (#3195T), N-cadherin (D4R1H) (#13116T), claudin-1 (D5H1D) (#13255T), β-catenin (D10A8) (#8480T), β-actin (#4967) (Cell Signaling Technology, Inc. (Danvers, MA, USA)), and STAT3 (#610190) (BD Biosciences, San Jose, CA, USA) at 1:1000 dilution; then the membranes were incubated overnight at 4 °C. Membranes were washed three times with TBS-N and then incubated with secondary antibody (mouse: #ab6789, rabbit: #ab6721) (both from Abcam, Cambridge, UK) for 30 min. After washing, the immunoblots were visualized using a chemiluminescence (ECL) HRP substrate (Bio-Rad, California, USA). The data were analyzed via densitometry using ImageJ software and normalized to the expression of the internal control (β-actin).

### 4.5. Wound Healing Assay

Cells (2 × 10^5^ cells/well) were seeded into a 24-well plate and allowed to adhere for 24 h. A 200 µL pipette tip was used to create a wound in the monolayer, after which the cells were washed twice with PBS and treated with asiatic acid in 1% FBS-containing media for 24 h. Cell migration was monitored under a light microscope at 0 and 24 h post-treatment.

### 4.6. Knockdown with siRNA

Cells were seeded into 60 mm dishes and allowed to grow to 80% confluence before transfection with siRNA using a Lipofectamine transfection reagent. A mixture of Lipofectamine ™ RNAiMAX (Thermo Fisher Scientific, Inc., Waltham, MA, USA), Opti-MEM™ (Invitrogen, NY, USA), and 10 nM of siRNA (Ctrl, STAT3 or claudin-1, all from Santa Cruz Biotechnology, Santa Cruz, CA, USA) was added with a complete medium for 24 h. To investigate the effect of asiatic acid on STAT3 and CLDN1 knockdown cells, the transfected cells were treated with DMSO or asiatic acid for 24 h. Cell viability and wound healing assays were then performed.

### 4.7. Statistical Analysis

The data are represented as mean ± standard error from at least three independent experiments. Statistical significance was determined using Student’s *t*-test, with *p*-values shown for * *p* ≤ 0.05, ** *p* ≤ 0.01, and *** *p* ≤ 0.001 when compared with control. One-way ANOVA followed by Tukey’s honestly significant difference test was also performed. *p* < 0.05 was considered statistically significant within a 95% confidence interval.

## Data Availability

Data is contained within the article and Supplementary Material.

## Supplementary Materials

The following supporting information can be downloaded at: https://www.mdpi.com/article/10.3390/ph16060902/s1, Figure S1: Asiatic acid inhibits constitutive STAT3 and IL-6-induced STAT3 phosphorylation in nasopharyngeal carcinoma cells.
