# Supplementary material for: Asiatic Acid Inhibits Nasopharyngeal Carcinoma Cell Viability and Migration via Suppressing STAT3 and Claudin-1
Source: Pharmaceuticals (Basel). 2023 Jun 19;16(6):902. doi: 10.3390/ph16060902 (PMC10301974; doi:10.3390/ph16060902)
Supplement: Supplementary file 1 [file pharmaceuticals-16-00902-s001.zip › pharmaceuticals-2422769-supplementary.pdf]

Supplementary Figure

A

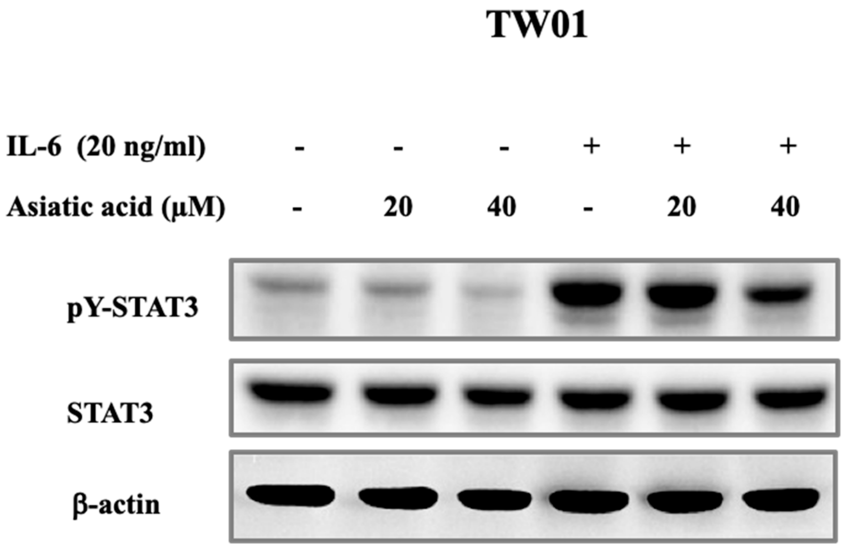

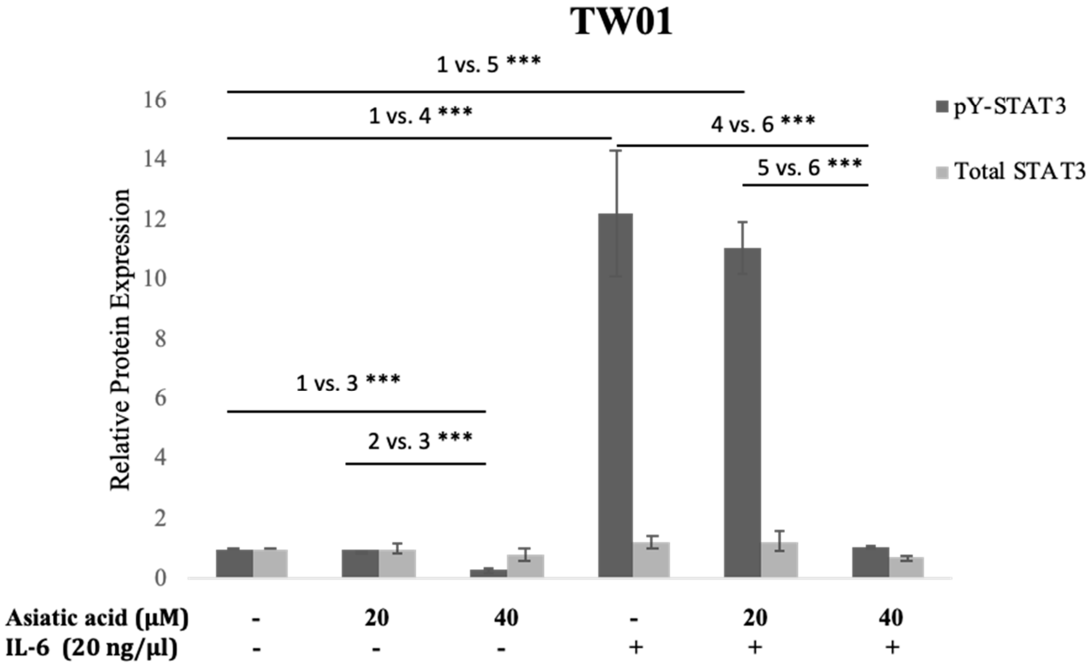

B

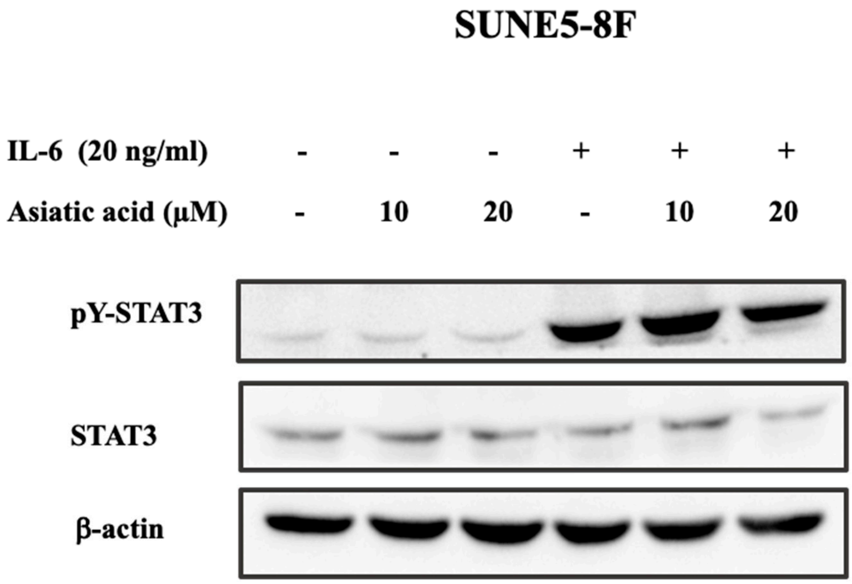

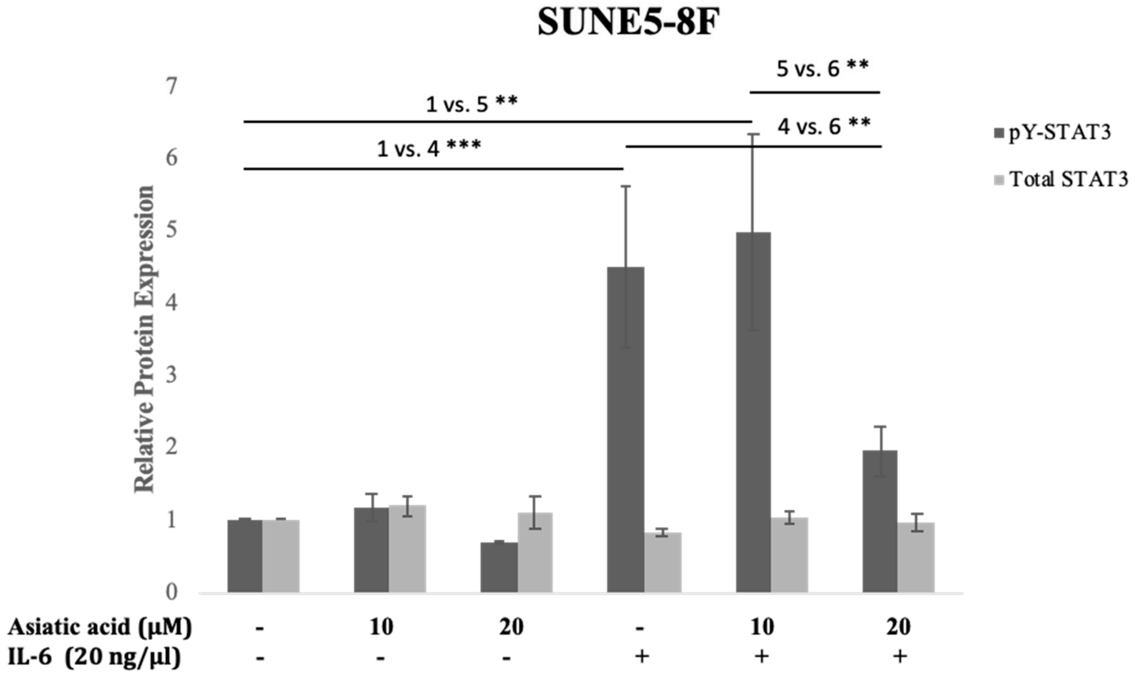

Figure S1. Asiatic acid inhibits constitutive STAT3 and IL-6-induced STAT3 phosphorylation in nasopharyngeal carcinoma cells. (**A**) TW01 and (**B**) SUNE5-8F cells were pretreated with asiatic acid at the indicated concentrations for 24 h and then stimulated with 20 ng/ml of IL-6 for 30 min. Western blot analysis was performed to examine the levels of phospho-STAT3 protein expression in TW01 and SUNE5-8F cells. β-actin was used as a loading control. Densitometry analysis of protein expression was quantified using ImageJ software. Data represented as mean ± SE from three independent experiments. * $p \le 0.05$, ** $p \le 0.01$, *** $p \le 0.001$ using one-way ANOVA followed by Tukey's honestly significant difference test.
